# The Perceived Neighborhood Crime and Hazardous Alcohol Use Among Youth in University of the Northeastern Thailand Context

**DOI:** 10.34172/jrhs.2020.33

**Published:** 2020-11-14

**Authors:** Suneerat Yangyuen, Suwimon Songklang, Udomsak Mahaweerawat, Chatchada Mahaweerawat

**Affiliations:** ^1^Faculty of Public Health, Mahasarakham University, Thailand; ^2^Faculty of Medicine, Mahasarakham University, Thailand

**Keywords:** Neighborhood, Alcohol, Youths, Thailand

## Abstract

**Background:** The residents’ perceptions of the crime and lack of safety with their neighborhood environment, associated with stress that confers risk for drinking.While many studies have focused on adult drinking, less is known about how subjective neighborhood crime influences drinking during adolescent. We aimed to determine the association of perceived neighborhood crime and youth alcohol use.

**Study design:** A cross-sectional study.

**Methods:** This study was conducted on 1087 university youths from 30 neighborhood clusters in Northeastern Thailand from May 2019 to Mar 2020.The data were collected by self-administered questionnaire.A multilevel logistic regression model was applied to examine the effect of perceived neighborhood crime on hazardous alcohol use.

**Results:**Most of youths were female, approximately 60.7 %reported hazardous alcohol use, and the average perceived neighborhood crime score was 65.1 (standard deviation, 2.1).The perceived neighborhood crime was associated with hazardous alcohol use; a 1-unit increase in the scores for perceived neighborhood crime corresponded to a 20 %increase in hazardous alcohol use. The role of perceived neighborhood crime on alcohol use varied among males, but not females.

**Conclusion:**The perceived neighborhood crime plays a role in the increase likelihood of hazardous alcohol use.The consideration of neighborhood crime context is important to design the alcohol preventive and intervention strategies.

## Introduction

Adolescent or youth drinking is significant public health concern. In 2016, the WHO reported approximately 26.5% of youth current drinking alcohol ^1^. In Thailand, alcohol use is one of the major issues facing Thai youth. Since 2018, the prevalence of youth current drinkers was 16.9%, and forecast that it will be increased from 16.2% in 2019 to 15.9% in 2020, and the second rank most prevalent found in the northeast region as 29.2% ^2^. Alcohol use is influenced by multiple social contexts, including neighborhood, family, and peers ^3^. Prior social epidemiology studies have shown that negative neighborhood factors such as neighborhood disorder, perceived neighborhood crime, or violence were related to increasing of alcohol use ^4-7^. In particularly, neighborhood crime, perception of crime and violence have been identified as chronic neighborhood stressors ^8,9^. Therefore, the residents exposure to crime or violence in their neighborhood can lead to stress, then alcohol may use to cope with stressful conditions^5,8,10^. Moreover, the role of neighborhood crime on substance use may differ by sex. Women perceived a greater risk of crime compared to men, while some study found the health effect of neighborhood context is larger for men ^11-13^.

However, despite the neighborhood crime may influence alcohol use, but there is no other study has conducted among youths and there are no statistics and evidence available on this subject in Thailand ^14^. Hence, investigating the effect of perceived neighborhood crime on youth alcohol use might be useful to develop prevention intervention strategies **.**

## Methods

###  Study population 

 This cross-sectional study was conducted in three universities located in upper-part (Udon Thani Province), middle-part (Mahasarakham province), and lower-part (Ubon Ratchathani province) of northeastern, Thailand from May 2019 to Mar 2020. The students aged 18-22 yr with no communication problems and resided in their neighborhood at least three months were considered as inclusion criteria in the study, while lack of interest to participate and incomplete questionnaires were introduced as extinction.

The calculation of the sample size was conducted using Cochran’s formula^15^, with estimator of the percentage of youths engage in hazardous alcohol use with brief behavioral counselling (35.5%) in the report followed by Bureau of Health Administration in 2018 ^16^ and a 95% confidence interval and desired precision of 3%. This accounted for 978 participants, then plus 10% compensation for nonresponse or dropout ^17^. The final sample size was 1087 from all 1,260 students enrolled and 173 students were excluded of incomplete response. Therefore, the 1087 students who met the eligible criteria were enrolled by multistage sampling technique.

 In the first stage, the three universities were selected using lottery method from the university geographical marked spot listings (one part per one university). In the second stage, the five faculties of each university were selected using lottery method from a list of faculties in each university. In the third stage, the students were selected by systematic random sampling in each university. Every fourth student was selected from the list and exclusion in case the student was absent or unwilling to take part in the research, and inclusion compensated from the student next on the list was taken in. All subjects were divided into 30 neighborhood clusters by the administrative unit of municipalities in each province.

 We stimulated a socio-ecological model, in which a multi-level framework is used to understanding the interaction between individuals and environmental factors in which they are embedded that we focused on the interplay among individual, interpersonal and neighborhood-level variables.

###  Instruments

 The self-administered questionnaire was composed of four parts as follows:

###  Covariates

Part 1: The individual-level variables included sex, age, and monthly house income, categorized as dichotomous variable. Besides, alcohol expectancies variable, assessed by alcohol expectancies scale (Aes) ^18^, reflecting expectations of a positive and negative effect of alcohol consumption. This scale comprised 15 items (eight items for positive alcohol expectancies (PAEs) and seven items for negative alcohol expectancies (NAEs)) and used a four-point scale, ranging from 1 (disagree) to 4 (agree). The total score was calculated by summing across all items of each dimension; PAEs rang 8-32 and NAEs rang 7-28, we dichotomize AEs by median. This scale has good internal consistency for both PAEs and NAEs (Cronbach’s alpha was 0.88 and 0.89, respectively). The content validity index (CVI) of PAEs and NAEs scale as 0.87 and 0.85, respectively.

 Part 2: The interpersonal-level variables were assessed by two items reflecting the extent to which peer and family members consumed alcohol.

Part 3: The neighborhood-level variable. We measured individual’s perception of their neighborhood crime during the past three months. This scale ^19,20^, included two parts: concern about crime (nine items) and neighborhood crime problems (nine items). The response for concern about crime ranged from 1 (strongly disagree) to 4 (strongly agree) on a four-point scale, while neighborhood crime problems ranged from 1 (rarely/not worried) to 10 (frequency/very) (Cronbach’s alpha was 0.84, indicating good internal consistency, and CVI as 0.83). We estimated the neighborhood-level crime score, by the mean of the individual’s total scores was calculated for each neighborhood, with higher mean scores indicating high perceived neighborhood crime.

###  Outcome variable

Part 4: The primary outcome of this study was alcohol consumption assessed through self-reported on current alcohol use. The respondents were asked whether or not they have ever used alcohol during the past 12 months, and we applied the alcohol use disorders identification test (AUDIT) (Thai version) to assess hazardous alcohol use. This scale was composed of 10 items and the total scores ranged from 0 to 40 (Cronbach’s alpha, 0.86 for the total scale, and CVI as 1.00). The total scores of 8 or more are considered to indicate hazardous alcohol use and total scores less than 8 as no hazardous alcohol use ^21^.

###  Statistical analysis

Descriptive analyses were performed for all variables characteristics. Next, a two-level multilevel binary logistic regression analysis via generalized linear mixed models was fitted to estimate the strength of the association between perceived neighborhood crime, each covariate, and alcohol consumption. The two-level structure comprised individuals at level 1 (including individual-level and interpersonal-level variables) nested within neighborhood at level 2. The model processing started with null model, and a series of the two-level model was developed. First, in model 1, include only individual-level variables into the model. Then, in model 2, all interpersonal-level variables were entered into model 1. Finally, in model 3, the perceived neighborhood crime variable was entered into model 2. The median odds ratio (mOR) and interval odds ratio (IOR) were applied for measure the variation of alcohol use in different neighborhoods and effect of neighborhood-level variable, respectively. Subsequently, to test differences by sex, we fit the same series of model 3 including a test of interaction between perceived neighborhood crime and sex (*P* for interaction <0.05). The statistically significant level was set at *P*<0.05 and SPSS software (Chicago, IL, USA) was performed for all analyses.

###  Ethical approval

 The written informed consent was obtained from each subject following the research information, conducted in accordance with the ethical principles and approved by the Review Ethics Broads of Mahasarakham University (Ethical no. PH056/2562).

## Results

Most of youths were female (51.2%), their median age was 19 yr old, and approximately two-thirds (60.7%) were hazardous drinkers. More than half (55.7%) of them had monthly household income 8,000 Thai baths or above (250 US$), and reported a high level of PAEs (56.3%) and NAEs (51.0%). Most respondents stated that their family members (52.4%) and friends (56.3%) used alcohol (Table 1).

###  Bivariate Models

These models indicated that the perceived neighborhood crime was significantly related to increased odds of hazardous alcohol use. Youths who were male, had a high level of PAEs and those whose family and peer consumed alcohol were more likely to have hazardous alcohol use patterns, whereas those who had a high level of NAEs were less likely to drinking (Table 2).

**Table 1:** Distribution of individual, interpersonal, and neighborhood-level variables by alcohol consumption.

**Variables**	**Total** **(n = 1087 )**		**Hazardous** **alcohol use (n = 660 )**		**No hazardous** **alcohol use (n = 427 )**	
	**Number**	**Percent**	**Number**	**Percent**	**Number**	**Percent**
**Individual - level**						
Sex						
Male	530	48.8	370	56.1	160	37.5
Female	557	51.2	290	43.9	267	62.5
Age (y)						
≥20	550	50.6	350	53.0	200	46.8
<20	537	49.4	310	47.0	227	53.2
Monthly household income (THB)						
≥8000	605	55.7	380	57.6	225	52.7
<8000	482	44.3	280	42.4	202	47.3
Positive alcohol expectancies						
High	612	56.3	415	62.9	197	46.1
Low	475	43.7	245	37.1	230	53.9
Negative alcohol expectancies						
High	554	51.0	300	45.5	254	59.5
Low	533	49.0	360	54.5	173	40.5
**Interpersonal - level**						
Family alcohol use						
Yes	570	52.4	400	60.6	170	39.8
No	517	47.6	260	39.4	257	60.2
Peer alcohol use						
Yes	612	56.3	428	64.8	184	43.1
No	475	43.7	232	35.2	243	56.9
**Neighborhood - level**	**Mean**	**SD**	**Mean**	**SD**	**Mean**	**SD**
Perceived neighborhood crime	65.1	2.1	65.5	2.2	64.2	1.8

**Table 2:** Odds ratios and 95% confidence intervals from multilevel binary logistic regression for hazardous alcohol use.

**Variables**	**Bivariate**		**Model 1**		**Model 2**		**Model 3**	
	**Unadjusted ** **OR (95%CI )**	***P*** ** value**	**Adjusted ** **OR (95%CI )**	***P*** ** value**	**Adjusted ** **OR (95%CI )**	***P*** ** value**	**Adjusted ** **OR (95%CI )**	***P*** ** value**
**Level - 1 **								
Gender								
Female	1.00		1.00		1.00		1.00	
Male	2.12 (1.66, 2.73)	0.001	1.99 (1.31, 3.02)	0.001	1.90 (1.27, 2.86)	0.002	1.81 (1.21, 2.71)	0.004
Age (yr)								
<20 y	1.00		1.00		1.00		1.00	
≥20	1.28 (1.00, 1.63)	0.046	1.30 (0.82, 2.05)	0.252	1.16 (0.73, 1.83)	0.525	1.15 (0.72, 1.81)	0.531
Monthly household income (THB)								
<8000	1.00		1.00		1.00		1.00	
≥8000	1.21 (0.95, 1.55)	0.114	1.18 (0.91, 1.52)	0.206	1.16 (0.88, 1.54)	0.292	1.12 (0.85, 1.49)	0.419
Positive alcohol expectancies								
Low	1.00		1.00		1.00		1.00	
High	1.98 (1.54, 2.53)	0.001	1.87 (1.37, 2.55)	0.001	1.97 (1.47, 2.65)	0.001	1.89 (1.43, 2.52)	0.001
Negative alcohol expectancies								
Low								
High	0.57 (0.44, 0.73)	0.001	0.61 (0.48, 0.78)	0.001	0.63 (0.49, 0.83)	0.001	0.63 (0.49, 0.84)	0.001
Using alcohol in family								
No	1.00		-		1.00		1.00	
Yes	2.33 (1.81, 2.98)	0.001	-	-	2.11 (1.71, 2.59)	0.001	2.09 (1.69, 2.58)	0.001
Using peer alcohol								
No	1.00		-		1.00		1.00	
Yes	2.44 (1.89, 3.13)	0.001	-	-	2.29 (1.81, 2.93)	0.001	2.26 (1.78, 2.87)	0.001
**Level - 2 **								
Perceived neighborhood crime								
No	1.00		-		-		1.00	
Yes	1.25 (1.17, 1.33)	0.001	-	-	-	-	1.20 (1.12, 1.29)	0.001
Random effects								
Level 1	-		1.00		1.00		1.00	
Level 2	-	-	0.20 (0.08, 0.49)	0.028	0.19 (0.07, 0.48)	0.034	0.08 (0.02, 0.35)	0.016

###  Multilevel Models

 The mOR in all models was greater than 1 (mOR for model 1 to 3 was 1.53, 1.51, and 1.31, respectively), which indicated that the between-neighborhood variation in alcohol use was greater than the within neighborhood-level variation, and the IOR-80% of neighborhood crime interval contained 1 (IOR-80%: 0.72, 2.01), which confirmed this finding further.

In model 1, revealed that the association between individual-level variables (i.e. sex, age, monthly house income, PAEs and NAEs) and hazardous alcohol use was similar to that of the bivariate model. In model 2, interpersonal-level variables were added to model 1, alcohol used by peer and family members were associated with an increased likelihood of hazardous alcohol use. In model 3, neighborhood-level variable was added into the model, and the results showed similar relationship between the individual-level and interpersonal-level variables with hazardous alcohol used as in model 2. In addition, each 1-unit increase in the perceived neighborhood crime score increased the likelihood of hazardous alcohol use by 20%, indicating that individuals who perceived high neighborhood crime were more likely to be current alcohol users (Table 2). Further, the perceived neighborhood crime on alcohol use varied among males, but not females. Male who perceived high neighborhood crime were 1.44 (95%CI: 1.24, 1.67) times more likely to drink alcohol compared to those who perceived low neighborhood crime (Table 3).

**Table 3:** Odds ratios and 95% confidence intervals between hazardous alcohol use and perceived neighborhood crime stratified by sex ^a^

**Variable**	**Male**		**Female**	
	**Adjusted** **OR (95%CI)**	***P*** ** value**	**Adjusted** **OR (95%CI )**	***P*** ** value**
Perceived neighborhood crime	1.44 (1.24, 1.67)	0.001	1.09 (0.96, 1.24)	0.150

^a^ All model adjusted for age, monthly household income, positive alcohol expectancies, negative alcohol expectancies, family and peer alcohol use

## Discussion

The finding showed that youths who perceived their neighborhood as more crime were more likely to engage in hazardous alcohol use, in accordance with other studies ^5,7,22^ that residents consume more alcohol when they perceive more neighborhood crime. An explanation of such finding is that neighborhood characterized by high level of disorders (e.g., high crime rate, perceived neighborhood crime, or violent crime) that may impact individual health behaviors through various mechanism, including physiological or psychological stress pathway ^23-25^. Especially, residing in a high crime neighborhood relates to fewer prosocial recreational activities and high availability of substances, including alcohol, and also could exposure multiple risks, which could accumulate as chronic stressors ^3,5,8,10^. Besides, neighborhood crime affecting an individual’s mental health disorders by increasing the risk of victimization and influencing residents’ perceptions of their disorder neighborhood as dangerous, threatening, or stressful^5,26^. Then, substance use like alcohol use may be one method to cope with neighborhood stressors such as neighborhood crime and violence^5,6,8,10^. Thus, the stress caused by living in a neighborhood with crime may be an important predictor of youths’ alcohol and drug use ^8,26^. Our findings are inconsistent with those of Tucker et al. ^4^ and Yabiku et al.^26^, potentially due to differences in the neighborhood measurements, study design, and study population.

Our findings, the perceived neighborhood crime on alcohol use differed for men and women suggest that women and men perceive their neighborhood crime differently, which can influence their stress responses and coping method ^6^. In addition, gender as a potential effect modifier between neighborhood exposure and health behaviors ^11,13^, which health effect of neighborhood context is larger in men. In particular, men residing in disordered neighborhoods may have greater opportunities to be associated with deviant peers or become involved in delinquent activities such as substance use ^27^.

Moreover, family members and peers drinking were associated with increased risk of youths’ alcohol use ^4,23,28,29^. In particular, deviant peers on substance use have a strong influence on alcohol use for youths residing in more disordered neighborhoods through alcohol offers and share positive attitudes toward alcohol use ^4,23,27,30^. Besides, addictive behaviors and substance use higher among families that have a history of substance involvement and members may be learning the substance use as a usual family pattern ^31^.

Our finding showed that PAEs was strongly associated with a greater chance of alcohol use; whereas, NAEs was inversely associated with drinking, which inconsistent with other studies ^32,33^. One possible explanation is that an individual's decision about whether or not to consume alcohol, is based on the anticipated positive and negative consequences associated with its use; PAEs is thought to promote alcohol use and relapse, whereas NAEs is thought to have the opposite effect ^34,35^. The alcohol expectancies can be obtained by observing parental or peers drinking behaviors and learning attitudes regarding alcohol use that due to youths were perceived benefits and visible effects of drinking behaviors^28,33,34^.

This study has some limitations. The self-reported alcohol use and perceived neighborhood crime can be implicated in recall bias and social durability bias. The minimization of self-report bias, validated and standardized instruments were used. Due to the cross-sectional design, so temporality and causality could not be inferred. Moreover, we used subjective measures of perceived neighborhood crime, which may provide different neighborhood characteristics from objective measures of crime. The perception of one’s neighborhood is stronger than objective measures in terms of relationship to health ^5,36^. Despite these limitations, our study has compensation strength to make a large sample size and control for a wide range of covariates. Our results provided further evidence of neighborhood-level risk factor regarding alcohol use among youths and revealed the importance of considering subjective neighborhood measures when investigating relations with health behavior, because to our knowledge; no other study has examined the effects of neighborhood crime on youth substance use in Thailand neighborhoods. Further, longitudinal studies are needed to evaluate the potential causal association between neighborhood effects act on youth substance use behaviors and objective measures of neighborhood crime should examine further.

## Conclusion

 Neighborhood crime influence on youth alcohol consumption. A better understanding of how neighborhood factors trigger alcohol use behavior is critical for developing interventions to prevent and reduce alcohol use. Besides, the differential sex finding suggested that the perceptions of neighborhood context are not uniform across population within neighborhoods, in which male drinkers who perceived high neighborhood crime areas are at increased odds of hazardous alcohol use. Therefore efforts to reduce alcohol drinking may have the most impact if targeted to a male subpopulation.

## Acknowledgements

 We are grateful to Faculty of Public Health, Mahasarakham University for research support funding and we would like to sincerely thank all study subjects for their participation.

## Conflict of interest

 There are no conflicts of interest.

## Funding

 We received grant support from Faculty of Public Health, Mahasarakham University.

## Highlights

- In Thailand, about two-thirds of youths reported hazardous alcohol use in the past-year.
- The perceived neighborhood crime influences the hazardous alcohol use among Thai youth.
- The role of perceived neighborhood crime on alcohol use varied among males.
